# Development and Validation of a Nomogram for Predicting Survival in Patients with Advanced Pancreatic Ductal Adenocarcinoma

**DOI:** 10.1038/s41598-017-11227-8

**Published:** 2017-09-14

**Authors:** Qing-Long Deng, Shu Dong, Lei Wang, Chen-Yue Zhang, Hai-Feng Ying, Zhao-Shen Li, Xiao-Heng Shen, Yuan-Bao Guo, Zhi-Qiang Meng, Jin-Ming Yu, Qi-Wen Chen

**Affiliations:** 1 Institute of Clinical Epidemiology, Key Laboratory of Public Health Safety of Ministry of Education, School of Public Health, Fudan University, 200032 Shanghai, China; 2 Department of Integrative Oncology, Shanghai Cancer Center, Fudan University, Shanghai, 200032 China; 3 Department of Oncology, Shanghai Medical College, Fudan University, 200032 Shanghai, China; 4 Digestive Endoscopy Center, Department of Gastroenterology, Changhai Hospital, Second Military Medical University, 200433 Shanghai, China; 5 Department of Integrative Medicine of Shanghai Ruijin Hospital, Shanghai Jiao Tong University School of Medicine, 200025 Shanghai, China

## Abstract

This study aimed to develop and validate an effective prognostic nomogram for advanced PDAC patients. We conducted a prospective multicenter cohort study involving 1,526 advanced PDAC patients from three participating hospitals in China between January 1, 2004 and December 31, 2013. Two thirds of the patients were randomly assigned to the training set (n = 1,017), and one third were assigned to the validation set (n = 509). Multivariate cox regression analysis was performed to identify significant prognostic factors for overall survival to develop the nomogram. Internal and external validation using C-index and calibration curve were conducted in the training set and validation set respectively. As results, seven independent prognostic factors were identified: age, tumor stage, tumor size, ALT (alanine aminotransferase), ALB (albumin), CA 19-9, HBV infection status, and these factors were entered into the nomogram. The proposed nomogram showed favorable discrimination and calibration both in the training set and validation set. The C-indexes of the training set and validation set were 0.720 and 0.696 respectively, which were both significantly higher than that of the staging system (C-index = 0.613, P < 0.001). In conclusion, the proposed nomogram may be served as an effective tool for prognostic evaluation of advanced PDAC.

## Introduction

Pancreatic ductal adenocarcinoma (PDAC), one of the most common malignant neoplasms of digestive system, is generally associated with poor prognosis and high mortality. The overall 5-year survival rate among PDAC patients is less than 5%^1^. In recent years, the morbidity of PDAC increased year by year. In 2010, the global incidence of PDAC was up to 292,471 cases^2^. According to *World Cancer Report 2014*, the United States had 46,420 (male: 23,530 vs female: 22,890) new cases of PDAC in 2014, and an estimated 39,590 patients died from this disease^3^. While in China, the new cases of 2015 were 90,100, and 79,400 patients died. The number of new cases ranked 9^th^ and deaths ranked 6^th^ among the 10 most common cancers. In addition, from 2000 to 2011, the morbidity and mortality of PDAC in China have taken on overall upward trends^4^.

It is known that prognostic evaluation is the basis of personalized cancer treatment. However, there are some intrinsic demerits in the traditional prognostic evaluation methods. TNM staging system is one of the most widely used methods in the prognostic evaluation of cancer; nevertheless, it only takes histologic metastasis of tumor into account and doesn’t incorporate many other important prognostic factors, such as age, gender, tumor size, and tumor marker. In this sense, the traditional staging system is an inaccurate method for prognostic evaluation. Therefore, a more accurate and comprehensive tool is needed. Nomogram is one such tool.

Nomogram is a visualization method of regression, it is primarily based on logistic and cox regression model. Compared to the traditional TNM staging system, nomogram can integrate more significant prognostic factors, which enables it to make more accurate evaluation. While compared to the traditional regression model, nomogram is more user-friendly due to the visualization function, and even clinicians lacking statistical expertise can easily read it. As a result, nomogram is being widely used in clinical prognostic evaluation, and may serve as a potential surrogate for the traditional staging system^5^.

In the field of PDAC, several nomograms have been already proposed. However, to our knowledge, a nomogram specific to Chinese PDAC patients, especially advanced patients, has not been reported. Besides, most of the proposed nomograms were constructed for specific population and derived from retrospectively collected data, so the clinical utility was limited. In this setting, the current study aimed to develop and validate a widely applicable prognostic nomogram for advanced PDAC patients via a large prospective multicenter cohort study in China.

## Patients and Methods

### Study population

From January 2004 to December 2013, we conducted a multicenter dynamic cohort study. Consecutive patients from three participating hospitals (Shanghai Cancer Center, Changhai Hospital, Ruijin Hospital) were prospectively included between January 1, 2004 and December 31, 2011. Inclusion criteria were as follows: pathologically proven PDAC; no history of other malignancies; treatment-naive patients of stage III and stage IV, who received palliative chemotherapy, and the first-line anticancer treatment was gemcitabine-based chemotherapy; met the requirements of follow-up and signed the informed consent. All the patients were first reviewed by two doctors at each participating hospital, and then the clinical data was transferred to Shanghai Cancer Center. Two experienced doctors at Shanghai Cancer Center went through these clinical materials to determine inclusion or exclusion of the patients. If divergent views arose, a third doctor would be consulted for the final assessment of inclusion or exclusion.

This study was carried on in accordance with the precepts of the Helsinki Declaration. Approvals were obtained from the Ethics Committee for Medical Research, School of Public Health, Fudan University. During the hospital stay, every included patient or their guardians signed the informed consent in view of prospective research of the clinical data.

### Data collection

Patient demographics (age and gender), smoking status, alcohol consumption, tumor stage, tumor size, tumor site, serum ALT (alanine aminotransferase), AST (aspartate aminotransferase), ALB (albumin), HBV infection status, and CA 19-9 were obtained at the diagnosis.

Based on the five indices in HBV profile (including HBsAg, HBsAb, HBeAg, HBeAb, HBcAb) and HBV-DNA level, HBV infection in this study was categorized into four statuses, namely, non infection, chronic HBV infection, inactive HBV carrier and resolved HBV infection. “Non infection” was defined as being negative for HBsAg and anti-HBc. “Chronic HBV infection” was defined as being HBsAg and anti-HBc positive, and either HBeAg positive or HBV DNA positive. “Inactive HBV carrier” was defined as HBsAg positive, and both HBeAg and HBV-DNA negative. “Resolved HBV infection” was defined as being HBsAg negative and either anti-HBe or anti-HBc positive^6, 7^.

### Follow up

The follow-up started at the time of diagnosis and ended when the patients were dead or censored. All the patients in this study were followed up regularly. Survival conditions were actively obtained once a month within the first year and then every 3 months thereafter. The follow-up ended on December 31, 2013.

### Statistical analysis

The statistical analyses were performed using R software version 3.3.2^8^ (R Development Core Team; http://www.r-project.org) with the survival, rms, and survivalROC package^9–11^. The significant level was set at 0.05 and all tests were two sided.

For nomogram construction and validation, we randomly assigned two thirds of the patients to the training set and one third to the validation set. The characteristics of the two sets were described and compared using chi-square test or one sample t test. Variables to develop the nomogram were selected by the stepwise selection method using Akaike information criterion (AIC) in the Cox proportional hazards (PH) model. The PH assumption was examined using Schoenfeld residual plots and multicollinearity was examined using variance inflation factor (VIF). Based on the predictive model with identified prognostic factors, a nomogram predicting median survival time (MST) and 1-year overall survival (OS) was constructed. After the construction of the nomogram, internal and external validation were performed in the training set and validation set respectively. Nomogram validation consisted of two parts, discrimination and calibration. Discrimination was evaluated using a concordance index (C-index). The value of the C-index ranged from 0.5 (no discrimination at all) to 1.0 (perfect discrimination). Calibration was performed by comparing the means of predicted survival with those of actual survival with observed Kaplan-Meier estimates after grouping of the nomogram predicted survival by decile. To reduce bias, bootstraps with 1,000 resamples were used for these activities. For clinical use of the model, the total points of each patient were calculated based on the proposed nomogram. Receiver operating characteristic curve analysis with censored survival data was performed to calculate the optimal cutoff values that were determined by maximizing the Youden index (ie, sensitivity + specificity − 1). Accuracy of the optimal cutoff value was assessed by the sensitivity, specificity, predictive values, and likelihood ratios. In addition, to further examine the discrimination of the proposed nomogram, we categorized the patients of the training set into four groups by the quartiles of nomogram total points and then calculated each group’s MST and plot each group’s Kaplan-Meier survival curve. These curves were compared using log-rank test.

### Data Availability

The dataset analyzed during this study is included in its Supplementary Information files.

## Results

### Cohort characteristics

In total, 1,526 consecutive patients with advanced PDAC were identified. In the training set (n = 1,017), the median follow-up was 6.8 months (range: 0.4 to 110.2 months). During this period, 912 patients (89.7%) died, and 105 patients (10.3%) were censored. The MST was 7.2 months (95%CI: 6.7 to 7.9 months). The 3-, 6- and 12-month survival rates were 84.2%, 57.3% and 27.2% respectively. While in the validation set (n = 509), the median follow-up was 6.7 months (range: 0.5 to 108.6 months). During this period, 450 patients (88.4%) died, and 59 patients (11.6%) were censored. The MST was 7.1 months (95%CI: 6.6 to 7.9 months). The 3-, 6- and 12-month survival rates were 86.3%, 58.1% and 30.8% respectively.

The patient characteristics were listed in Table 1. There were no significant differences among the following variables between the training set and validation set.

**Table 1:** Characteristics of the training set and validation set.

	Total (n = 1,526)	Training set (n = 1,017)	Validation set (n = 509)	Statistics	P value
Age (years), n (%)				0.008	0.927
<60	815 (53.4)	544 (53.5)	271 (53.2)		
≥60	711 (46.6)	473 (46.5)	238 (46.8)		
Gender, n (%)				0.027	0.870
Male	973 (63.8)	647 (63.6)	326 (64.0)		
Female	553 (36.2)	370 (36.4)	183 (36.0)		
Smoking, n (%)				0.500	0.480
Yes	733 (48.0)	482 (47.4)	251 (49.3)		
No	793 (52.0)	535 (52.6)	258 (50.7)		
Alcohol, n (%)				0.002	0.964
Yes	212 (13.9)	141 (13.9)	71 (14.0)		
No	1,314 (86.1)	876 (86.1)	438 (86.0)		
Tumor stage, n (%)				1.376	0.241
III	595 (39.0)	386 (38.0)	209 (41.1)		
IV	931 (61.0)	631 (62.0)	300 (58.9)		
Tumor site, n (%)				1.256	0.262
Head and neck	644 (42.2)	419 (41.2)	225 (44.2)		
Body and tail	882 (57.8)	598 (58.8)	284 (55.8)		
Tumor size (cm)				−0.362	0.717
Median	5.9	5.9	5.9		
Range	0.8-15.0	1.6–15.0	0.8–12.0		
ALT, n (%)				1.519	0.218
Normal	1,301 (85.3)	859 (84.5)	442 (86.8)		
Elevated	225 (14.7)	158 (15.5)	67 (13.2)		
AST, n (%)				0.085	0.770
Normal	1,353 (88.7)	900 (88.5)	453 (89.0)		
Elevated	173 (11.3)	117 (11.5)	56 (11.0)		
ALB, n (%)				0.289	0.591
Normal	1,226 (80.3)	821 (80.7)	405 (79.6)		
Low	300 (19.7)	196 (19.3)	104 (20.4)		
CA 19–9, n (%)				0.551	0.458
Normal	325 (21.3)	211 (20.7)	114 (22.4)		
Elevated	1,201 (78.7)	806 (79.3)	395 (77.6)		
HBV infection status, n (%)				2.632	0.452
Non infection	833 (54.6)	543 (53.4)	290 (57.0)		
Inactive HBV carrier	83 (5.4)	56 (5.5)	27 (5.3)		
Resolved HBV infection	549 (36.0)	373 (36.7)	176 (34.6)		
Chronic HBV infection	61 (4.0)	45 (4.4)	16 (3.1)		
Center, n (%)				3.654	0.161
Shanghai Cancer Center	833 (54.6)	539 (53.0)	294 (57.8)		
Changhai Hospital	486 (31.8)	331 (32.5)	155 (30.4)		
Ruijin Hospital	207 (13.6)	147 (14.5)	60 (11.8)		

### Independent prognostic factors in the training set

The results of multivariate cox regression were listed in Table 2. Older age (≥60 vs. <60, P < 0.001), stage IV (vs. III, P < 0.001), larger tumor size (P < 0.001), elevated ALT (vs. normal, P < 0.001), low ALB (vs. normal, P < 0.001), and elevated CA 19-9 (vs. normal, P < 0.001) were associated with poor prognosis. As for HBV infection status, chronic HBV infection (P = 0.007) was associated with better prognosis compared to non infection.

**Table 2:** Multivariate cox regression of the training set.

	β	HR	HR 95% CI	Z value	P value
Age (years)					
<60	Ref*	Ref	Ref	—	—
≥60	0.43	1.53	1.33–1.75	6.089	<0.001
Tumor stage					
III	Ref	Ref	Ref	—	—
IV	0.80	2.22	1.93–2.56	11.025	<0.001
Tumor size (cm)	0.07	1.07	1.03–1.11	3.881	<0.001
ALT					
Normal	Ref	Ref	Ref	—	—
Elevated	0.63	1.87	1.57–2.24	6.920	<0.001
ALB					
Normal	Ref	Ref	Ref	—	—
Low	0.69	1.98	1.68–2.34	8.169	<0.001
CA 19-9					
Normal	Ref	Ref	Ref	—	—
Elevated	0.65	1.92	1.63–2.28	7.600	<0.001
HBV infection status					
Non infection	Ref	Ref	Ref	—	—
Inactive HBV carrier	−0.28	0.76	0.56–1.02	−1.838	0.070
Resolved HBV infection	0.30	1.35	1.17–1.55	4.090	<0.001
Chronic HBV infection	−0.47	0.63	0.46–0.85	−2.946	0.003

^*^Ref = Reference group; HR = Hazard Ratio; CI = Confidence Interval.

### Prognostic nomogram for MST and OS

**Figure 1:**
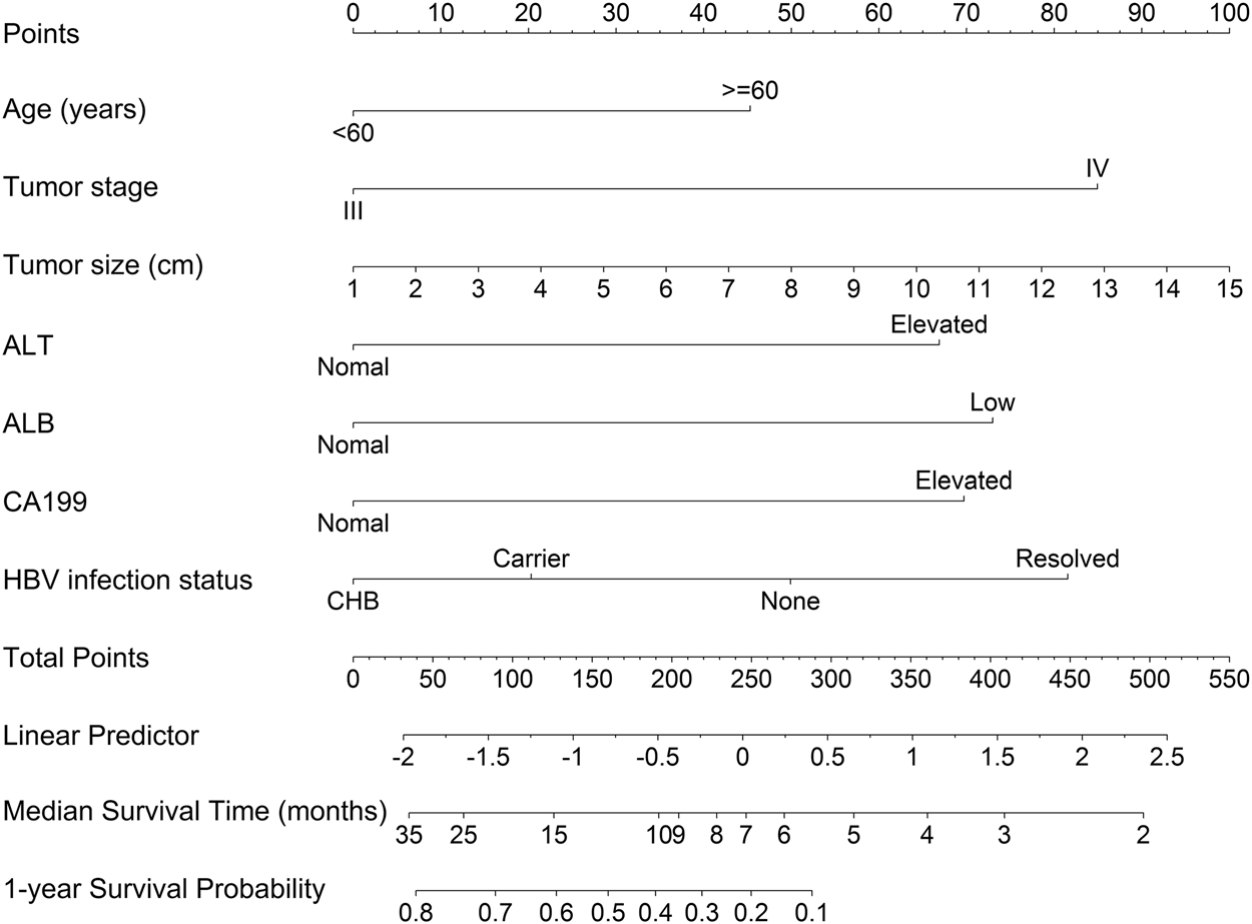
Nomogram for predicting median survival time and 1-year survival probability of advanced PDAC patients. (To use the nomogram, an individual patient’s value is located on each variable axis, and a line is drawn upward to determine the number of points received for each variable value. The sum of these numbers is located on the Total Points axis, and a line is drawn downward to the survival axes to determine the estimated median survival time and 1-year survival probability). Abbreviations: CHB = Chronic HBV infection, Carrier = Inactive HBV carrier, None = Non infection, Resolved = Resolved HBV infection.

The prognostic nomogram that integrated all the variables selected by multivariate cox regression was shown in Fig. 1. The nomogram illustrated tumor size as sharing the largest contribution to prognosis, followed by tumor stage, HBV infection status, ALB, CA 19-9, ALT, and age. Each subtype within these variables was assigned a score on the point scale. By adding up the total score and locating it on the total point scale, we can easily draw a straight line down to determine the estimated median survival time and 1-year survival probability.

### Validation of the nomogram

**Figure 2:**
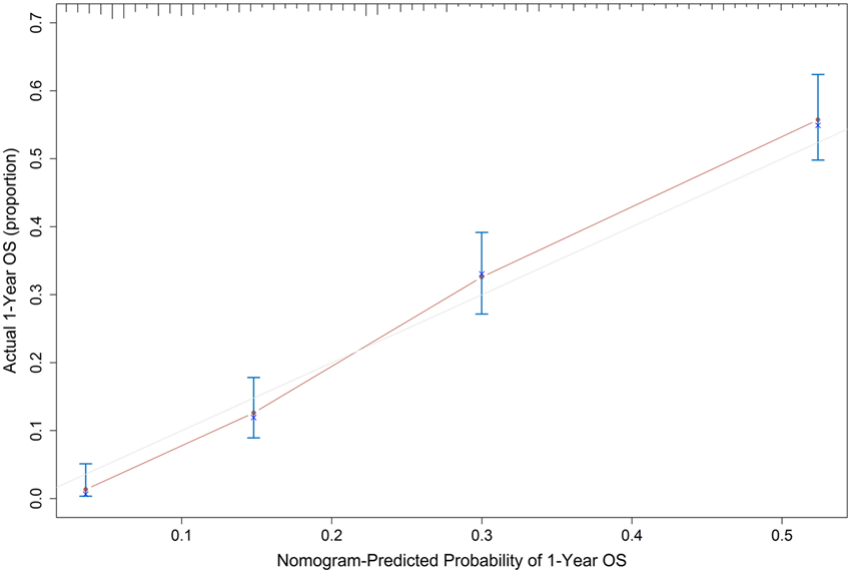
The calibration curve for predicting 1-year survival probability of advanced PDAC patients in the training set.

**Figure 3:**
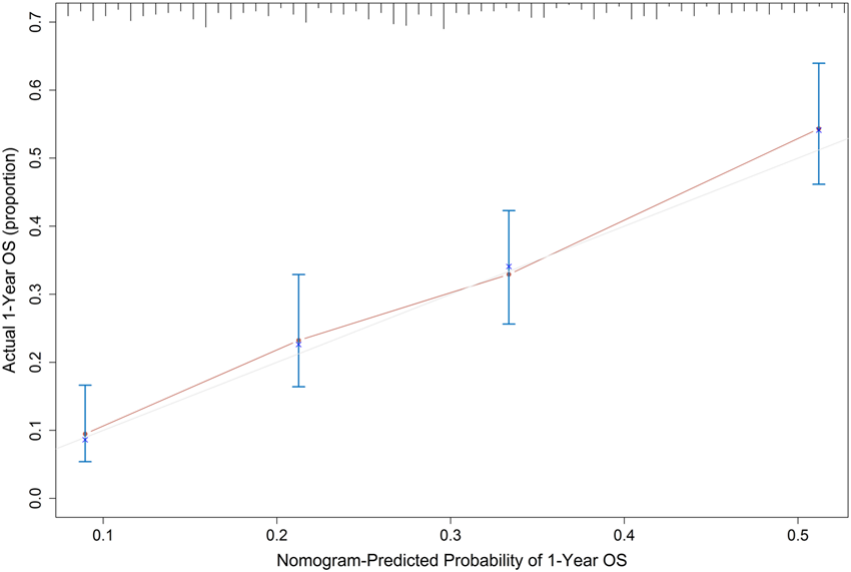
The calibration curve for predicting 1-year survival probability of advanced PDAC patients in the validation set.

The calibration curves for predicting 1-year survival probability in the training set and validation set were shown in Fig. 2 and Fig. 3. Both of the curves showed good agreement between the nomogram prediction and actual observation for 1-year OS.

In the training set, the C-index was 0.720 (95%CI: 0.703 to 0.737), which was significantly higher than that of the staging system (C-index = 0.613, 95%CI: 0.597 to 0.629; P < 0.001). In the validation set, the C-index was 0.696 (95%CI: 0.670 to 0.722), which was also significantly higher than that of the staging system (P < 0.001). The bias-corrected C-indexes in the training set and validation set were 0.716 and 0.696 respectively.

The area under ROC curve (AUC) of the training set and validation set were 0.792 and 0.732 respectively. The optimal cutoff value of nomogram total points was determined to be 216 in the training set and 214 in the validation set. The sensitivity, specificity, positive predictive value, negative predictive value, positive likelihood ratio, and negative likelihood ratio for predicting 1-year survival probability were as listed in Table 3.

**Table 3:** Accuracy of the proposed nomogram for predicting 1-year survival probability.

Variable	Training set	Validation set
Area under ROC curve (AUC)	0.792	0.732
Cutoff point	216	214
Sensitivity, %	77.1	77.3
Specificity, %	71.1	56.8
Positive predictive value, %	95.9	93.2
Negative predictive value, %	26.3	24.7
Positive likelihood ratio	2.67	1.79
Negative likelihood ratio	0.322	0.400

**Figure 4:**
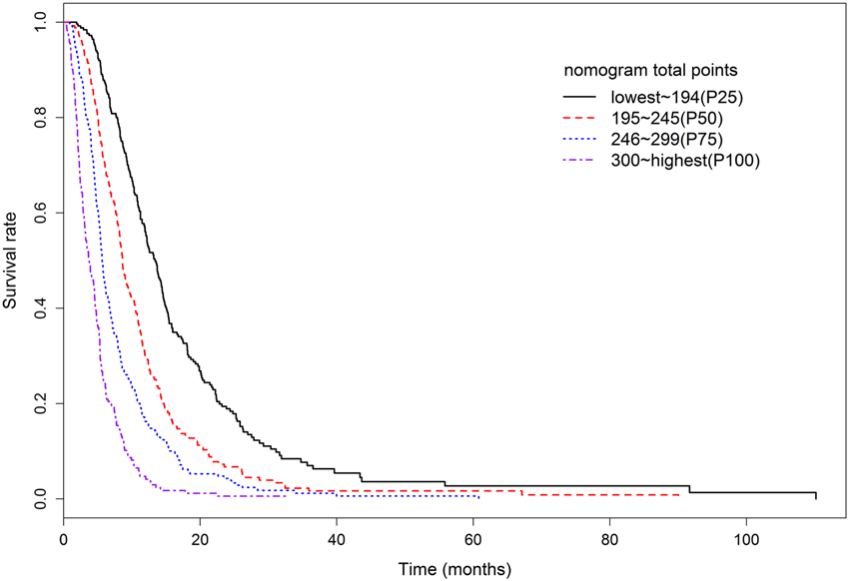
Kaplan-Meier survival curves of training set categorized by the quartiles of proposed nomogram total points.

To determine the performance of the proposed nomogram in stratifying risk of patients, we categorized the patients of the training set into four subgroups based on the quartiles of nomogram total points (ie, lowest to 194, 195 to 245, 246 to 299, 300 to highest). The MST of each group was 13.4 months (95%CI: 12.0 to 14.4 months), 8.6 months (95%CI: 8.2 to 9.5 months), 5.7 months (95%CI: 5.4 to 6.2 months), 3.7 months (95%CI: 3.2 to 4.5 months), respectively. Fig. 4 illustrated the Kaplan–Meier survival curves according to the nomogram-based groupings. The survival times were significantly differentiated between the subgroups (P < 0.001).

## Discussion

Pancreatic ductal adenocarcinoma is heterogeneous in regard to survival of individual patients; therefore, prognostic evaluation solely based on the traditional staging system is imprecise. Despite several previously reported nomograms among PDAC patients, a nomogram has not been developed for Chinese advanced PDAC patients. Thus, we sought to develop and validate such a prognostic nomogram to predict overall survival of this population. Favorable discrimination and calibration could be found in the proposed nomogram derived from prospectively collected data on 1526 patients from three hospitals in China. Superior to the existing TNM staging system, nomogram would facilitate the popularization of patient counselling and personalized treatment. Additionally, the proposed nomogram was constructed with a large prospective multicenter cohort study, rendering it more widely applicable than the previous reported nomograms.

In this study, we identified seven independent prognostic factors for advanced PDAC, namely, age, tumor stage, tumor size, ALT, ALB, CA 19-9, and HBV infection status. Our previous study^12^ revealed that HBV status is a significant factor affecting the progression of advanced PDAC, and that chronic hepatitis B infection may be a protective factor for these patients. As is generally recognized, long-term persistence of HBV infection can cause an inflammatory microenvironment in the liver^13^, which may trigger enhanced immune defense^14^. The boosted immune responses were assumed to be beneficial in the inhibition of tumor progression among PDAC patients. In addition, HBV replication may boost tumor necrosis factor α (TNF-α) secretion via regulating hepatocytes and immune cells residing in the liver^15^. In view that HBV can also replicate in pancreas^16^, we speculate that HBV also plays a role in the biological behavior of PDAC, thereby attenuating tumor invasiveness. These underlying effects altogether prolonged overall survival of PDAC patients. This result of our study was consistent with Qian HG *et al*.’s study^17^ among colorectal cancer patients. However, Wei *et al*.^18^ found that HBV infection increased synchronous liver metastasis incidence. The prognostic role of HBV infection for advanced PDAC patients and the underlying mechanisms need to be further explored via rigorously designed RCTs and biomedical experiments.

CA 19-9 was first isolated from a colorectal cell line and has since become the most widely used tumor marker for PDAC^19^. Because serum CA 19-9 level is intimately associated with the overall tumor burden of PDAC, patients with elevated CA 19-9 level may be at more advanced stage, compared to those without elevated CA 19-9^20, 21^. Perioperative CA 19-9 level has been studied to determine the efficacy from radical surgery^22–24^. Several studies reported that preoperative CA 19-9 was linked with resectability and postoperative prognosis^25–27^. Moreover, postoperative CA 19-9 level can predict overall survival and disease-free survival after cancer resection and adjuvant chemotherapy^28, 29^. Our study confirmed the results from other studies. Saad *et al*.^30^ found that pretreatment CA19-9 level was an independent predictor of OS in PDAC patients who once had received gemcitabine chemotherapy. On the contrary, in a study with the sample size of 67 patients, Sezgin *et al*.^31^ identified performance status was the only independent prognostic factor of OS in locally advanced or metastatic PDAC patients who had undergone gemcitabine treatment and CA 19-9 level had nothing to do with the treatment response to gemcitabine. Whether CA 19-9 could be served as a reliable and applicable prognostic marker of advanced PDAC needs to be verified in large cohort studies. With around 10-year follow-up and a larger sample size, the current multi-center study suggested that CA 19-9 was a confirmative independent prognostic factor for advanced PDAC.

In addition to serum CA 19-9, decreased ALB level was found as a poor prognostic factor for advanced PDAC in this study. Serum ALB is the most abundant blood protein in mammals. For advanced PDAC, decreased ALB level often means relatively later stage of the disease, which can be caused by insufficient intake of nutrition, malabsorption, and accelerated decomposition of food^32^. Clinically, ALB supplementation during the disease course may improve nutrition status and thus help extend survival of advanced PDAC patients, but this needs to be verified by well-designed clinical trials.

Serum ALT and AST are commonly used as clinical indicators for liver function^33^. However, they have been rarely taken as prognostic factors for advanced PDAC. Our study suggested that elevated ALT level was a poor prognostic factor for advanced PDAC. It may be explained that elevated ALT reflects the consequences of cancer cell’s invasion into the liver and liver injury. Most of the time, without liver metastasis or liver injury, ALT or AST may not elevate. Thus, maintenance of ALT and AST levels to the normal ranges could prolong survival of advanced PDAC patients.

Besides the mentioned factors above, age at diagnosis, tumor stage, and tumor size were also found to be associated with prognosis of advanced PDAC. Most previous studies^34–36^ illustrated the prognostic roles of these factors in advanced PDAC and the results of our study were consistent with their findings.

There are also some limitations in the present study. Firstly, we randomly divided the patients into two subgroups, 2/3 for construction and 1/3 for validation of the nomogram. Although this is a generally accepted method of nomogram build and validation, yet it is still an alternative when external cohort is not available. Hence, our proposed nomogram needs to be validated in other population derived from an external cohort. Secondly, not all the potential prognostic factors are included in the nomogram, so it can’t make absolutely accurate prediction. However, the results of validation demonstrated good fitness of the present nomogram based on the seven variables for survival prediction. Thirdly, tumor stage was included into the nomogram as dichotomous variable (stage III or IV) rather than subdivision of TNM stage, which may result in less accuracy for prediction. Fourthly, none of included patients received FOLFIRINOX. FOLFIRINOX is a combination chemotherapy regimen consisting of oxaliplatin, irinotecan, fluorouracil, and leucovorin, which has been shown to prolong survival of patients with metastatic pancreatic adenocarcinoma compared to gemcitabine as first-line therapy^37^. FOLFIRINOX was first published in 2011, while our study started at the year of 2004 and gemcitabine-based chemotherapy was the standard regimen at that time. Besides, FOLFIRINOX is relatively expensive and patients who receive FOLFIRINOX may experience more severe side effects than those who receive gemcitabine alone, so this combination is usually given to patients who are healthy enough to tolerate the potential side effects^37, 38^. Therefore, FOLFIRINOX is not widely used for the treatment of advanced pancreatic cancer in China. In spite of this, FOLFIRINOX may become a prospective and promising chemotherapy for PDAC in the coming future in China because of its notable treatment response. Accordingly, we are undertaking to take it into clinical practice and planning to further study this regimen in future research.

In conclusion, the proposed nomogram can accurately predict overall survival of patients with advanced PDAC. Compared to the traditional staging system, clinicians can promote individual-oriented cancer therapy and disease management by using this tool.

## Electronic supplementary material

Dataset 1
